# Supplementary material for: Chromothripsis during telomere crisis is independent of NHEJ, and consistent with a replicative origin
Source: Genome Res. 2019 May;29(5):737–49. doi: 10.1101/gr.240705.118 (PMC6499312; doi:10.1101/gr.240705.118)
Supplement: Supplemental Material [file supp_gr.240705.118_Supplemental_file_1.zip › contigs/annotated_contigs/DB93/contig.2.DB93_length_649_mean_cov_9.01386748844.docx]

**DB93_length_649_mean_cov_9.01386748844**

```
GAAGTCCAGAGTGGTAAAATCACATCATGGAGCCATTGGGCCAATGTTACTTCTGTACTTTTGCCCAATCATCCTTACCCTGTGGGTAT
 >chr3:128403861-128404183 + E=5e-182
CATCCTCATTCTTGTCACCTCAGGGTCATAAGATGGCTGTTACCTCCTCTATCACATCCGCATTTCAGACAGGGTAGAAGGGAAGAGAA

AATGGCAAACCATACTTGCCAGCTCAGTCAGCCCTTGTTTTTATGAACTTTTCAGGAAGCCCCACCCAGTAGCTTCTGCCTACACTTCA

TTACCTAGAACCATATTATATTACTTGGCCACTTCTCCCTCCAAGGAACAC|TGAG|TTTTTTTAAAAAGCAAATTCAGTGTAATGCTA
                                                    >chr6:12084320-12084651 + E=2e-181
TGGCAACTCTCTCAGCTTTGTCAGTGCTGGTTATTCTGGTCAAGCTTTCCTCATTCTGGATCAGTTTGGGTAGTTGTGTGTTTTTCTAC

GATTTTACTAGTTCATCACAATTTTCAAATTCATTAGCACAGAGTTGAGTCTTTGATTTCTTTTTCACTTACTGCAGGCAGTGTTTTTG

TTTGAAATATTGCTAGTCTAGACTATCATTTTCAGCATTATAACCGCGTGTCATCTTCCTCAAAGACAGGTAAGTCCAGCGGTATTTGA

GATGTGAAACTCGGAGGATAAAATGGGT
```
